# Prospective Evaluation of Infection Episodes in Cancer Patients in a Tertiary Care Academic Center: Microbiological Features and Risk Factors for Mortality

**DOI:** 10.4274/tjh.2015.0216

**Published:** 2016-12-01

**Authors:** Nursel Çalık Başaran, Ergun Karaağaoğlu, Gülşen Hasçelik, Mine Durusu Tanrıöver, Murat Akova

**Affiliations:** 1 Hacettepe University Faculty of Medicine, Department of Internal Medicine, Ankara, Turkey; 2 Hacettepe University Faculty of Medicine, Department of Biostatistics, Ankara, Turkey; 3 Hacettepe University Faculty of Medicine, Department of Basic Microbiology, Ankara, Turkey; 4 Hacettepe University Faculty of Medicine, Department of Infectious Diseases and Clinical Microbiology, Ankara, Turkey

**Keywords:** Febrile neutropenia, Cancer, mortality, risk factors

## Abstract

**Objective:**

We aimed to determine the frequency, type, and etiology of infections and the risk factors for infections and mortality in hospitalized cancer patients.

**Materials and Methods:**

We prospectively enrolled adult cancer patients hospitalized in the internal medicine wards of a tertiary care academic center between January and August 2004. Patients were followed during their hospitalization periods for neutropenia, infections, culture results, and mortality.

**Results:**

We followed 473 cancer patients with 818 hospitalization episodes and 384 infection episodes in total. Seventy-nine percent of the infections were nosocomial, and febrile neutropenia (FN) was observed in 196 (51%) of the infection episodes. Bacteremia was found in 29% of FN episodes and in 8% of nonneutropenic patients. Gram-positive bacteria were the leading cause of bacteremia in both neutropenic and nonneutropenic cases (70% and 58%, respectively). Presence of an indwelling central catheter increased bacteremia risk by 3-fold. The overall mortality rate was 17%, whereas 34% of the patients with bloodstream infections died. Presence of bacteremia and advanced disease stage increased overall mortality by 6.1-fold and 3.7-fold, respectively.

**Conclusion:**

Nearly half of the cancer patients developed an infection during their hospital stays, with gram-positive bacteria being the predominant etiologic microorganisms. This demonstrates the changing trends in infections considering that, until 2004, gram-negative bacteria were the most predominant microorganisms among cancer patients in our institute.

## Abstract (tr)

**Amaç:**

Enfeksiyonlar, kanser hastalarında önde gelen morbidite ve mortaliteleri nedeni olmuşlardır. Bu çalışmada hastanede yatan kanser hastalarında enfeksiyonların sıklığını, tiplerini, etiyolojilerini ve enfeksiyon gelişimi ve mortalite için risk faktörlerini belirlemeyi amaçladık.

**Gereç ve Yöntemler:**

Üçüncü basamak bir üniversite hastanesinin iç hastalıkları servislerinde Ocak-Ağustos 2004 tarihleri arasında izlenmiş olan erişkin kanser hastaları dahil edildi. Yatış süreleri boyunca nötropeni, enfeksiyonlar, kültür sonuçları ve mortalite açısından prospektif olarak izlendiler.

**Bulgular:**

Toplam 473 kanser hastasının 818 hastaneye yatış atağı izlendi. Toplam 818 yatış atağı sırasında 384 (%46) enfeksiyon atağı gözlendi- %79’u nozokomiyaldi. Febril nötropeni (FN) tüm atakların 196’sında (%51) görüldü. Bakteremi, FN ataklarının %29’unda ve nötropenik olmayan hastaların %8’inde görüldü. Gram-pozitifler hem nötropenik olan hem de olmayan hastalardaki bakteremilerin önde gelen etkeni olarak görüldü (%70 ve %58, sırasıyla). Santral kateter varlığının bakteremi riskini 3 kat artırdığı görüldü. Toplam mortalite %17 iken bakteremisi olan hastalarda mortalite %34 saptandı. Bakteremi varlığı ve ileri evre hastalık toplam mortaliteyi, sırasıyla, 6,1 ve 3,7 kat artırmaktaydı.

**Sonuç:**

Hastanede yatan kanser hastalarının neredeyse yarısında en azından bir enfeksiyon gelişmektedir ve bu enfeksiyonlarda gram-pozitifler hakimdir. Bu bilgiler, 2004 yılına kadar kanser hastalarında en sık görülen mikroorganizmaların gram-negatif mikroorganizmalar olduğu göz önüne alındığında, enfeksiyonlarda değişen eğilimler olduğunu göstermektedir.

## INTRODUCTION

Infections have become the leading cause of mortality and morbidity of cancer while supportive and curative treatment strategies prolong life [1,2]. Cancer and its treatment suppress the immune system, and long and recurrent hospitalizations predispose patients to various infections.

Predominant infectious pathogens have been variable in time with changing cancer treatment strategies, antibacterial prophylaxis practices, and emerging resistance patterns in bacteria. Until the 1980s, the leading microorganisms in cancer patients were enteric gram-negative bacteria and Pseudomonas aeruginosa. As of the 1980s, gram-positive bacteria became the most common pathogens in these patients [1,3,4]. However, recently, nonfermenting gram-negative bacteria have emerged as the leading pathogens in cancer patients.

It is important to know the risk factors for infections, changing epidemiology, and resistance patterns of pathogenic microorganisms for the proper management of infections in cancer patients. In this study, we aimed to determine the frequency, type, and etiology of infections and the risk factors for infections and mortality in hospitalized cancer patients.

## MATERIALS AND METHODS

### Study Design and Patients

This study was done in the internal medicine wards of a tertiary care university hospital. The institutional review board approved the study and adult cancer patients hospitalized between January and August 2004 were enrolled and followed prospectively. Demographic data, cancer type and stage, previous stem cell transplantation history and type, comorbidities, and presence of antibacterial utilization in the previous month were recorded upon admission. Presence of indwelling catheters (central or peripheral venous, arterial, urinary, or drainage), presence of parenteral nutrition, requirement of intensive care unit and mechanical ventilation, therapy for cancer (chemotherapy, radiation, corticosteroids), vital signs, infections, antibiotic usage, culture results, and neutrophil counts were recorded throughout the admission episode. Descriptive data and further analyses were done based on the admission episodes unless otherwise specified. One patient might have had more than one admission. The infectious diseases department followed the patients and the researchers did not intervene in the diagnostic and therapeutic processes.

Neutropenia was defined as an absolute neutrophil count below 500/mm3 or below <1000/mm3 and expected to decline rapidly. Neutropenic infections were classified as clinically or microbiologically documented infection, bloodstream infection (BSI), or fever of unknown origin [5]. Infections were classified as nosocomial according to the 1998 definitions of the Centers for Disease Control and Prevention [6]. Metastatic solid tumors, newly diagnosed hematological malignancies with poor prognosis, and relapsed or treatment-resistant hematological malignancies were defined as advanced disease stage. Corticosteroid use was defined as the use of prednisolone at a dose of >20 mg/day (or equivalent) or over a period of 10 days whatever the dose was. Antifungal prophylaxis was defined as oral fluconazole/itraconazole used in prophylactic doses.

### Microbiological Methods

All the cultures were collected from different parts of the body according to the presumed infections. They were inoculated onto suitable media and incubated at 37 °C for 24-48 h. Catheter cultures were studied quantitatively. For blood cultures, a BD BACTEC 9000 Blood Culture System (Becton Dickinson Diagnostic Systems, Sparks, MD, USA) was used. All the microorganisms were identified by gram staining, conventional microbiological tests (such as hemolysis, catalase, oxidase, and coagulase reaction), and the Phoenix System (Becton Dickinson Diagnostic Systems). Antibiotic susceptibility tests were conducted with the Phoenix System and for Streptococcus pneumoniae by E-test (AB BIODISK, Solna, Sweden). Results were evaluated according to the Clinical and Laboratory Standards Institute 2004 standards.

### Statistical Analysis

Data were analyzed by SPSS 11.5 for Windows (SPSS Inc., Chicago, IL, USA). Distribution of data was analyzed by Kolmogorov-Smirnov test. Normally distributed data are presented as mean ± standard deviation, while abnormally distributed data are presented as median (minimum-maximum). Categorical variables were compared by chi-square test and Fischer’s exact test where appropriate, and continuous variables were analyzed by Student’s t-test. Risk analysis was performed by Fisher’s exact chi-square test and parameters that were found to be significant were introduced into a multivariate logistic regression model. Relative risk was computed for possible risk factors with 95% confidence interval and p<0.05 was accepted as statistically significant.

## RESULTS

During the study period, 473 cancer patients between 16 and 82 years of age were enrolled and 818 hospitalization episodes were followed prospectively. Of these patients, 286 (60%) were male and the mean age was 51 years (16-82 years). Chronic diseases accompanying admission episodes were as follows: 4.8% coronary artery disease, 2.7% chronic renal failure, 10.2% diabetes mellitus, and 13.4% hypertension. Solid organ cancer was seen in 254 (53%) patients while the remaining had hematological diseases (Table 1). Hematopoietic stem cell transplantation (HSCT) was done in 49 patients with 63 admission episodes (7.7%) and half of them were allogeneic HSCTs.

In the course of 818 hospitalization episodes, a total of 384 (46%) infection episodes were observed and 79% of these were nosocomial. Febrile neutropenia (FN) was observed in 126 patients having 196 (51%) infection episodes. Acute myeloid leukemia was the most common underlying disease (n=35, 35/126, 27%) in patients with FN. Mean duration of neutropenia was longer in patients with an infection (16.2 days) when compared to those without an infection (8.2 days) (p=0.002). Bacteremia was found in 29% of FN episodes and in 8% of nonneutropenic infections (p<0.05). Sites of infections in neutropenic and nonneutropenic patients are shown in Table 2. The mean hospitalization duration was three times longer for patients with infection (38±31 days) when compared to the mean of the total hospitalization episodes (13±23 days).

Fluconazole as antifungal prophylaxis was given in 63 (7.7%) episodes as a part of the stem cell transplantation regimen. Corticosteroids were used in 215 (26.4%) of the admission episodes. Radiation therapy was performed in 5.1% (42) of hospitalization episodes. Unfortunately, we had no data about granulocyte colony-stimulating factor use in this study.

Nonneutropenic episodes constituted 71.4% of all the hospitalization episodes and in 77.3% of these cases an immunosuppressive treatment, including corticosteroids, was used. As expected, there was an immunosuppressive treatment in 94% of neutropenic episodes (p<0.001). Among the nonneutropenic episodes, infections were more frequently seen in those patients receiving any immunosuppressive treatment than in episodes with no immunosuppressive treatment (p<0.001). However, there was no difference in terms of mortality (p=0.111).

At least one pathogenic microorganism was isolated from culture specimens obtained from patients during 187 (48.6%) infection episodes. Blood cultures were positive in 29.6% of all the patients and in 60.6% of neutropenic patients. Gram-negative microorganisms were the most common (51%) isolates among all the specimens, whereas gram-positive microorganisms were the most common (65%) among blood culture isolates (Table 3). Fungi were isolated in 5% of all the specimens and 9% of the specimens from neutropenic patients. When bacteremic episodes were considered, gram-positive bacteria were the leading cause in both neutropenic and nonneutropenic cases (70% and 58%, respectively; p<0.05) (Table 4).

The resistance patterns of Staphylococcus aureus, Staphylococcus epidermidis, Escherichia coli, Klebsiella spp., and Pseudomonas spp. are listed in Table 5. In neutropenic patients, rates of extended-spectrum β-lactamase (ESBL)-producing E. coli and Klebsiella spp. were found to be high compared to nonneutropenic patients (p<0.05).

Possible risk factors for infection in cancer patients were analyzed by univariate analysis and then the risk factors found to increase the occurrence of an infection were introduced into a multivariate logistic regression model (Table 6). We found that advanced disease stage, neutropenia for more than 7 days, and radiation were related to an increased frequency of infection in cancer patients (p<0.05). Presence of an indwelling central catheter increased bacteremia risk by 3-fold (Table 6).

The overall mortality rate was 17%, whereas 34% of patients with BSIs died (p<0.05). Among patients with FN, the mortality rate was 18.4%, and the occurrence of a BSI increased the mortality rate to 41%. Presence of bacteremia increased overall mortality 6.1 times and advanced disease stage increased overall mortality 3.7 times. On the other hand, usage of prophylactic antifungal therapy decreased mortality 3.3-fold, but the p-value was found to be statistically insignificant (p=0.055) (Table 6). Comorbid chronic diseases had no significant effect on infection or mortality.

## DISCUSSION

This study is a landmark study to show the shift of infectious etiologies in cancer patients from gram-negative to gram-positive bacteria. Afterwards, a long-term multicentric study was established in Turkey for microbiological surveillance of FN patients. Such surveillance studies are valuable to implement systemic changes in individual institutions and countries.

In this prospective observational study we found that 46% of all cancer patients developed at least one infection and 85% of neutropenic patients developed at least one FN attack during their index hospital stay. Among the neutropenic attacks, 65% were documented clinically or microbiologically, and the BSI rate was 29% among the FN attacks, comparable to that reported in numerous other studies [5,7,8,9]. Compared to previous surveillance data from our hospital, both documented clinical infection and BSI rates were increased, which might be attributed to increased awareness of FN and appropriate blood culture techniques [10,11]. The definition of BSI may also have an influence on infection rates; the criteria used for skin flora organisms to be pathogens may be a reason for increased BSI rate.

Methicillin-resistant coagulase-negative staphylococci (MR-CoNS) were the most commonly isolated microorganisms from overall and blood culture specimens. The predominance of gram-positive bacteria and MR-CoNS in BSIs in this cohort was parallel to findings in the literature. In cancer patients BSIs were due to gram-negative enteric bacteria and Pseudomonas aeruginosa in the 1960s and 1970s, but by the middle of the 1980s gram-positive bacteria had become predominant [12,13]. Memorial Sloan-Kettering Cancer Center reported that the incidence of gram-positive BSIs increased from 14% to 23% between 1977 and 1987 [1,8]. During the same time period, both the European Organisation for Research and Treatment of Cancer (EORTC) and the Febrile Neutropenia Study Group reported that 55%-60% of BSIs were due to gram-positive microorganisms [14]. In 2003, Wisplinghoff et al. reported that in cancer patients nosocomial BSIs in 32% of neutropenic cases and 33% of nonneutropenic cases were due to CoNS [3]. Srinivasan et al. reported that in stem cell transplantation recipients, gram-positive bacteria, and especially members of skin flora such as Staphylococcus epidermidis, were predominant in BSIs [9]. Mikulska et al. reported the gram-positive to gram-negative ratio as 60%:40% in BSI infections of cancer patients [15]. In our study, the MR-CoNS ratio was the ratio between the blood cultures, so repetitive isolations from a patient should be kept in mind. We also accepted a CoNS as a pathogen when ≥1 blood culture was positive in the presence of fever or hypothermia, hypotension, indwelling catheter, or antibiotics, which could differ from other studies in the literature [1].

Recent studies revealed that in hematological malignancies gram-negative microorganisms have again become the most relevant microorganisms in BSIs. Cattaneo et al. reported a predominance of gram-negative bacteria (57.3%) in hematological malignancies between 2004 and 2010 [16]. Gudiol et al. reported that 49% of BSIs in hematological malignancies were due to gram-negative microorganisms and it was concluded that gram-positive microorganisms had decreased after quinolone prophylaxis [17]. A recently published paper by Trecarichi et al. also reported the shift from gram-positive to gram-negative bacteria in BSIs in hematologic malignancies and again they pointed out the increasing resistance among gram-negative bacteria [18]. Several studies demonstrated gram-negative predominance either in blood or other specimen cultures in hematologic or solid cancer patients, with a frequency ranging between 24.7% and 75.8% in different geographic places with high resistance rates, including ESBL-positive Enterobacteriaceae, multidrug-resistant Pseudomonas aeruginosa, Acinetobacter spp., and Stenotrophomonas maltophilia [19,20,21,22]. According to surveillance data between 2005 and 2009 from our institution, gram-negative bacteria became the predominant BSI etiology in hematological malignancies with high resistance patterns [23]. Our study differs from these other studies in two major points: first, in our study, we followed both hematological and solid cancer patients, and second, we accepted at least one positive culture with CoNS in the presence of fever or central venous catheter. The growing resistance problems, especially among gram-negative pathogens, require special efforts in infection control measures and rational antibiotic usage in cancer patients.

In this study ESBL-positive E. coli (55%) and Klebsiella spp. (50%) were more frequent in neutropenic cases than nonneutropenic cases. A literature review revealed that ESBL positivity in cancer patients ranged from 12% to 75% for E. coli and K. pneumoniae in different studies [23,24,25,26,27,28,29]. It was also shown that ESBL positivity negatively affects mortality and morbidity [26,30,31]. Unfortunately, due to low case numbers, we could not analyze the mortality effect of resistant gram-negative bacteria.

We found that patients who were neutropenic for 7 or more days were prone to infection 3.9-fold more so than others. Poor prognosis and advanced stage solid or hematologic cancers were also related to an increased infection risk by 3.1-fold. Radiation was another risk factor for infection. This might be explained by the characteristics of the patient group that received radiotherapy: poor performance status, palliation in advanced disease, advanced age, or total body radiation prior to stem cell transplantation.

Indwelling central catheter was a risk factor for BSIs. In the last 30 years, increased use of persistent indwelling catheters has brought about an increased infection risk, especially for CoNS BSIs [1,12,32,33]. Moreover, BSI was a risk factor for mortality in our study. In previous studies mortality in cancer patients with BSIs ranged between 20% and 35% and this changed according to the pathogenic microorganisms [21,34,35,36,37,38]. This also points to the importance of implementing catheter bundles to decrease catheter-associated BSI rates.

Antifungal prophylaxis was part of the prophylaxis regimen in HSCT patients and it seemed to lower the mortality, although we could not show statistical significance. There are some reports showing azole-resistant breakthrough fungemia, but a recent study from the EORTC revealed that antifungal prophylaxis was protective in fungemia in cancer patients [39]. As there are various studies on different oral antifungal prophylaxes with different outcomes favoring posaconazole, itraconazole, or fluconazole use in high-risk patients, further studies are required about which drug to use for which patient and how long these drugs must be used [40,41,42].

## CONCLUSION

Nearly half of the cancer patients developed an infection during their hospital stays, with gram-positive bacteria being the predominant etiologic microorganisms. This demonstrates the changing trends in infections considering that, until 2004, gram-negative bacteria were the most predominant microorganisms among cancer patients in our institute. Each patient must be evaluated individually for risk factors, and while antibiotic treatment is being planned, current local surveillance data and the resistance patterns of the microorganisms should be taken into account along with individual risk factors.

## Acknowledgment

A part of this study was presented as a poster presentation at the Febrile Neutropenia Symposium, February 2005, Ankara, Turkey, and the Interscience Conference on Antimicrobial Agents and Chemotherapy, December 2005, Washington, DC, USA.

## Ethics

Ethics Committee Approval: LUT 05/15; Informed Consent: It was taken.

## Figures and Tables

**Table 1:**
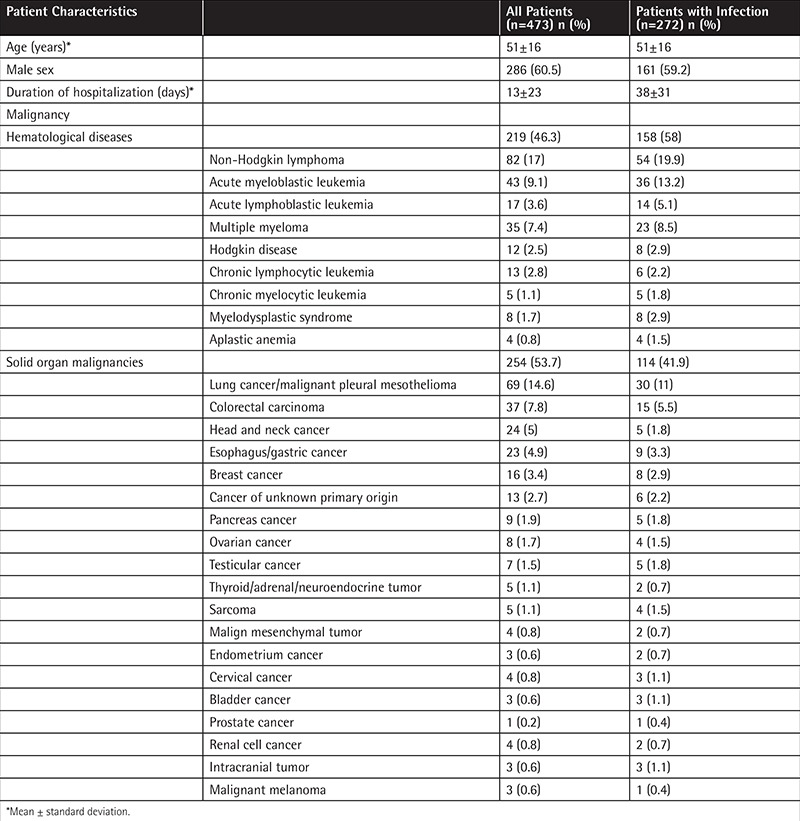
Demographic features of all patients and particularly patients with an infection.

**Table 2:**
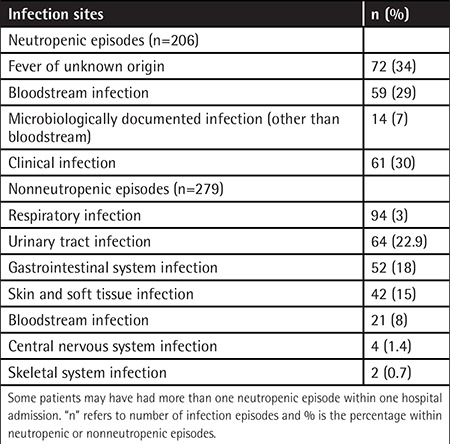
Distribution of infection sites in neutropenic and nonneutropenic patients.

**Table 3:**
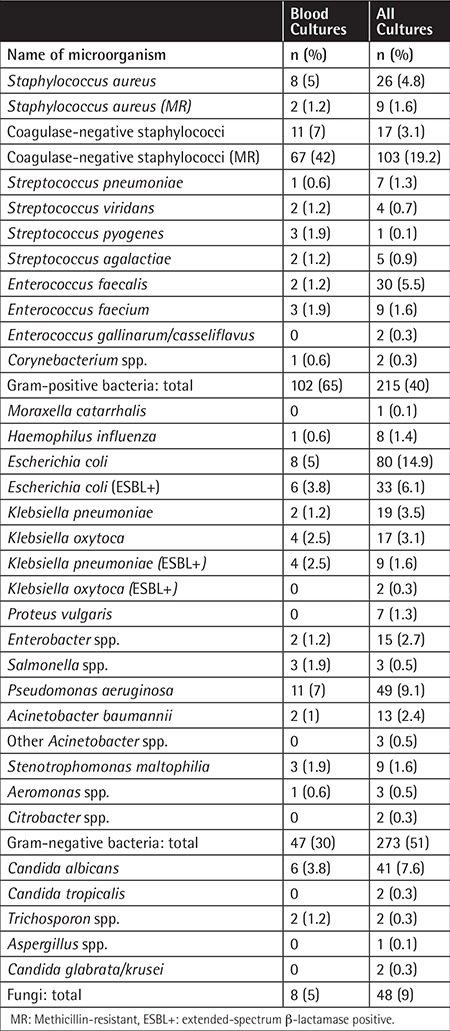
The results of blood cultures and all cultures.

**Table 4:**
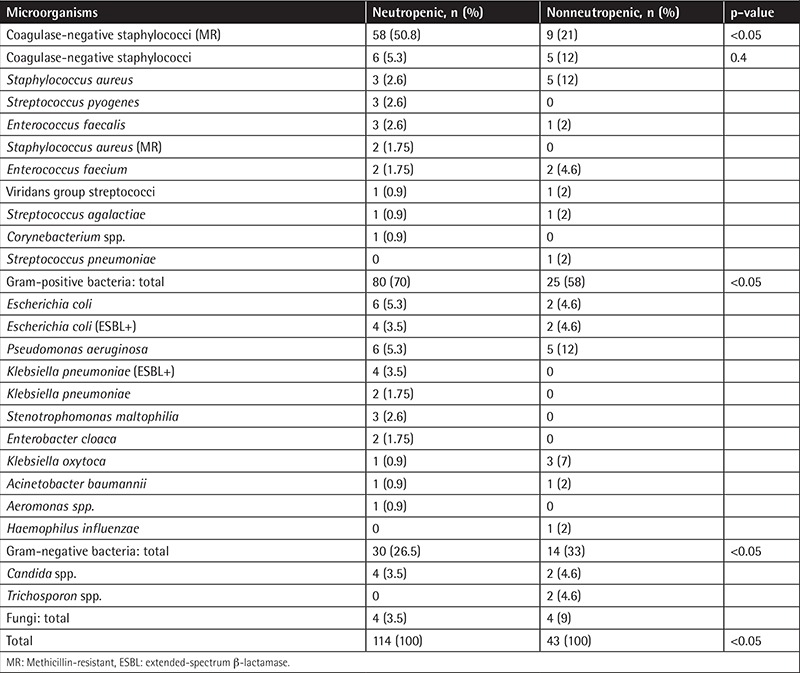
Isolates of blood cultures in neutropenic and nonneutropenic patients.

**Table 5:**
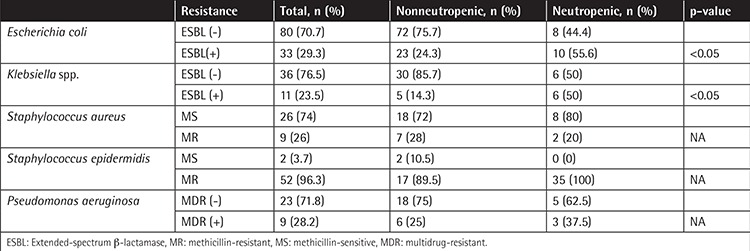
Resistance patterns of microorganisms in neutropenic and nonneutropenic patients.

**Table 6:**
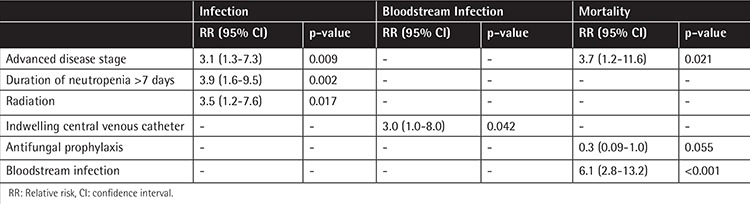
Risk factors for infection, bloodstream infection, and mortality.
